# DO OLDER ADULTS APPRAISE ELDERSPEAK COMMUNICATION AS PATRONIZING OR RESPECTFUL IN HOSPITAL DEMENTIA CARE?

**DOI:** 10.1093/geroni/igad104.2981

**Published:** 2023-12-21

**Authors:** Clarissa Shaw, Caitlin Ward, Lisa Weimar

**Affiliations:** University of Iowa, Iowa City, Iowa, United States; University of Minnesota, Minneapolis, Minnesota, United States; University of Iowa, Iowa City, Iowa, United States

## Abstract

Elderspeak communication is speech that sounds like babytalk to older adults. The Iowa Coding Scheme for Elderspeak (ICodE) contains 11 elderspeak attributes: diminutives, childish terms, collectives, short words, directives, reflectives, minimizers, exaggerated praise, laughter, tag questions, and exaggerated prosody. Thirty-one older adults (aged 74.5±7.8 years) listened to 41 clips of nursing staff providing care to hospitalized patients with dementia and rated each clip using the 5-point Emotional Tone Rating Scale for patronizing and respectful (5=very, 1=not at all). Each clip was classified using the ICodE as either neutral (no elderspeak), containing a single attribute, or containing an attribute with exaggerated prosody. Linear mixed modeling was used to evaluate the differences in patronizing and respectful ratings between neutral clips and each attribute, and clips with each attribute and exaggerated prosody, while controlling for within-subjects correlation and adjusting for multiple comparisons. Mean ratings for neutral clips were 4.0±1.1 for respectful and 1.7±1.0 for patronizing. Ratings for elderspeak attributes ranged from 2.1 to 2.9 for patronizing and 2.8 to 3.8 for respectful. Ten of the 11 attributes, excluding exaggerated praise, were rated as significantly more patronizing and less respectful than neutral. The addition of exaggerated prosody was significant in comparison with the elderspeak attribute alone for exaggerated praise (more patronizing and less respectful), laughter (more patronizing), short words (less patronizing, more respectful), and diminutives (less respectful). This analysis validates that the attributes of the ICodE are considered more patronizing and less respectful by older adults compared to neutral communication in hospital dementia care.

